# Lead Screening for Chronic Obstructive Pulmonary Disease of IKK2 Inhibited by Traditional Chinese Medicine

**DOI:** 10.1155/2014/465025

**Published:** 2014-06-02

**Authors:** Yung-An Tsou, Hung-Jin Huang, Wesley Wen-Yang Lin, Calvin Yu-Chian Chen

**Affiliations:** ^1^Otolaryngology Head and Neck Surgery, China Medical University Hospital, Taichung 40402, Taiwan; ^2^School of Medicine, College of Medicine, China Medical University, Taichung 40402, Taiwan; ^3^Department of Chinese Pharmaceutical Sciences and Chinese Medicine Resources, College of Pharmacy, China Medical University, Taichung 40402, Taiwan; ^4^Department of Biological Science and Technology, National Chiao Tung University, Hsinchu 30010, Taiwan; ^5^Department of Biomedical Informatics, Asia University, Taichung 41354, Taiwan

## Abstract

Chronic obstructive pulmonary disease (COPD) is a chronic obstructive lung disease and is frequently found in well-developed countries due to the issue of aging populations. Not all forms of medical treatment are unable to return a patient's limited pulmonary function back to normal and eventually they could require a lung transplant. At this time, COPD is the leading cause of death in the world. Studies surveying I-kappa-B-kinase beta (IKK2) are very relevant to the occurrence and deterioration of the condition COPD. The sinapic acid-4-O-sulfate, kaempferol, and alpha-terpineol were found to be IKK2 inhibitors and helped prevent COPD occurrence and worsening according to a screening of the traditional Chinese medicine (TCM) database. The protein-ligand interaction of these three compounds with regard to IKK2 was also done by molecular dynamics. The docking poses, hydrogen bond variation, and hydrophobic interactions found Asp103 and Lys106 are crucial to IKK2 binding areas for IKK2 inhibition. Finally, we found the three compounds that have an equally strong effect in terms of IKK2 binding proven by the TCM database and perhaps these may be an alternative treatment for COPD in the future.

## 1. Introduction

COPD, a chronic obstructive pulmonary disease, can lead to the restriction of lung function [1, 2]. The current treatment options for COPD are very limited and the side effect of treatment frequently noted is Cushing syndrome caused by long term steroid use [3]. Many COPD patients finally need lung transplants and the survival outcome is still poor even when patients undergo lung transplants [4]. Even with improvement in regard to pharmacy and drug invention techniques, the occurrence of COPD and mortality related to COPD continues to rise [5]. Clearly, efforts to prevent smoking, reduce air-pollution, and control pneumonia could be the appropriate prevention methods to limit deterioration in cases of COPD. However, there are no other useful ways to attempt to cure the COPD; thus it remains the leading cause of death throughout the world [2, 6]. Therefore, prevention of the occurrence of COPD is the important issue to address, by not only the above mentioned methods but also the inhibition of I-kappa-B-kinase beta (IKK2) which is linked to COPD occurrence [7–10].

IKK2 activation is related to many inflammatory diseases, severe immune compromise diseases [11], severe skin infection [12, 13], chronic intestinal infection [14], synovial inflammation [15], arthritis [16], pancreatitis, and diabetes mellitus [17]. There have been other reports that highlighted IKK2 activation as leading to chronic airway diseases such as Lipopolysaccharides (LPS) related airway infections, asthma, emphysema, and chronic bronchitis [7, 18]. The very important subunit of I*κ*B kinase is IKK2 that has an enzyme function that is involved in activating the NF-kB (nuclear transcription factor kappa-B) and causing the subsequent inflammatory condition IKK2 could be phosphorated by a protein called the I*κ*B (I*κ*B*α*), which could inhibit the NF-*κ*B by direct binding. Ubiquitination process is then started after I*κ*B phosphorylation, and then I*κ*B is degraded and set free of NF-*κ*B into the nucleus and causes subsequent immune and inflammation responses [16, 17, 19].

Utilizing a computational simulation technique could efficiently help identify suitable drugs for IKK2 inhibition through the use of computer-aided drug design (CADD) which can help to structure the best drug candidates and predict biological activity. CADD is a very efficient way to treat any specific disease with appropriate drugs targeting [20]. The novel targeting factors for drug design should be based on some related studies [21–27] and also include risk factor analysis [28–32]. This method is also advantageous in terms of economic efficiency [33]. The drug design by its structure and ligand-based binding survey are two essential components of CADD [34–38]. Then, the appropriate drug candidates are screened by molecular simulation that are structure-based and confirm the molecular dynamics in CADD [39].

Traditional Chinese medicine (TCM) has been used in China, Taiwan, Korea, and Japan for thousands of years. The largest traditional Chinese medicine database thus far is the TCM Database@Taiwan (http://tcm.cmu.edu.tw/) [39] which contains 61,000 compounds of Chinese herbal medicine with their 2D and 3D chemical structures, molecular information, and bioactivity. Novel lead compounds have been found for the treatment of cancer [40, 41], controlling pain [42], and virus detoxification [33, 34, 43] by TCM Database@Taiwan since 2011. The TCM Database@Taiwan is an important tool that will help to determine TCM drug design [21, 44–51] to help overcome related clinical difficulties and it could be accessed by the website portal [33] or through the cloud computing platform [52].

Based on recent study, a possible lead compound for COPD treatment has been identified by the TCM Database@Taiwan in this study. The docking screening of selected COPD ligands is done by utilizing computational techniques and confirming the molecular dynamics (MD) for protein-ligand interactions affected the most regarding IKK2 inhibitions in COPD.

## 2. Materials and Methods

### 2.1. Data Set

Molecular simulations were performed by Accelrys Discovery Studio 2.5 (DS 2.5) system. There were 61,000 TCM compounds downloaded from TCM database (http://tcm.cmu.edu.tw/) analyzed in this study. The IKK2 (PDB ID: 4KIK) [53] crystal structure from the RCSB Protein Data Bank was used for research, and the staurosporine was used as a control.

### 2.2. Molecular Dockings

LigandFit [54] was used to dock SC-514 staurosporine and TCM compounds to IKK2 in the CHARMm force field for docking simulation [55]. LigandFit is a program set on Discovery Studio 2.5 (DS 2.5) for docking receptor-rigid algorithm. The IKK2 docking site was surveyed and the top 3 compounds according to the docking score were obtained and Ligplot plus program was used for their hydrophobic interactions analysis [56, 57]. After that, we evaluated the protein-ligand interaction and drug efficacy by comparing the disorder region and the docking site.

### 2.3. Detection of the Disordered Protein

Disorder region was predicted according to protein structure and docking site by the Database of Protein Disorder (DisProt, http://www.disprot.org/). Thus, we could prevent disorder effects on drug design and determine the docking site and the efficacy of the drug more precisely [54, 58].

### 2.4. Simulation of Molecular Dynamics

The surveyed ligands prepared for further MD simulation were supported by SwissParam (http://swissparam.ch/) [55] on the basis of reference force field [56] counted by GROMACS 4.5.5 [57]. The docked ligands and IKK2 protein complex were set into the simulation box in the buffer. The minimum distance was set at 1.2 Å from the complex in the cubic box and then solvated in TIP3P water circumstances in which the complex charge was neutralized by adding sodium and chloride. The steepest descent method was used for minimizing complex for 5000 steps. The structure after minimization was finally used for MD simulation. The particle-mesh Ewald (PME) method was then used for calculating the electrostatic interactions between the ligands and IKK2 complex [59]. Each step was set for 2 fs and 2,500,000 steps were performed by the PME method. The Berendsen weak thermal coupling method was used for equilibration under constant temperature (NVT ensemble) for 100 ps was performed. The total time was set to 5000 ps for MD simulation process. Then the protocol in Gromacs was finally applied for MD trajectories, RMSD, and energy variations of complex were surveyed.

## 3. Results and Discussions

### 3.1. Docking Results for Molecules

The top 3 TCM compounds were selected (Table 1) by molecular docking according to their docking scores. These TCM compounds are sinapic acid-4-O-sulfate, kaempferol, and alpha-terpineol belonging to the TCM herbs, the bark of* Magnolia officinalis*,* Bupleurum chinense*, and* Bursaphelenchus xylophilus*. The first compound, sinapic acid-4-O-sulfate, has an antidepressant like effect [60, 61] and the herb, the bark of* Magnolia officinalis*, has potent anti-inflammation effects to prevent further tissue inflammation [62, 63]. The 2nd compound, the herb* Bupleurum chinense*, can be hepatoprotective, anti-inflammatory, analgesic, and antipyretic and also prevent acute lung injury and just be a good treatment material for COPD [64]. The 3rd compound alpha-terpineol and herb* Bursaphelenchus xylophilus* have an antimicrobial effect and in particular prevent infections that originate from periodontopathic and cariogenic bacteria [65]. Most of these compounds can prevent humans from further infection and inflammation and could be protectors for lung injury.

**Figure 1:**
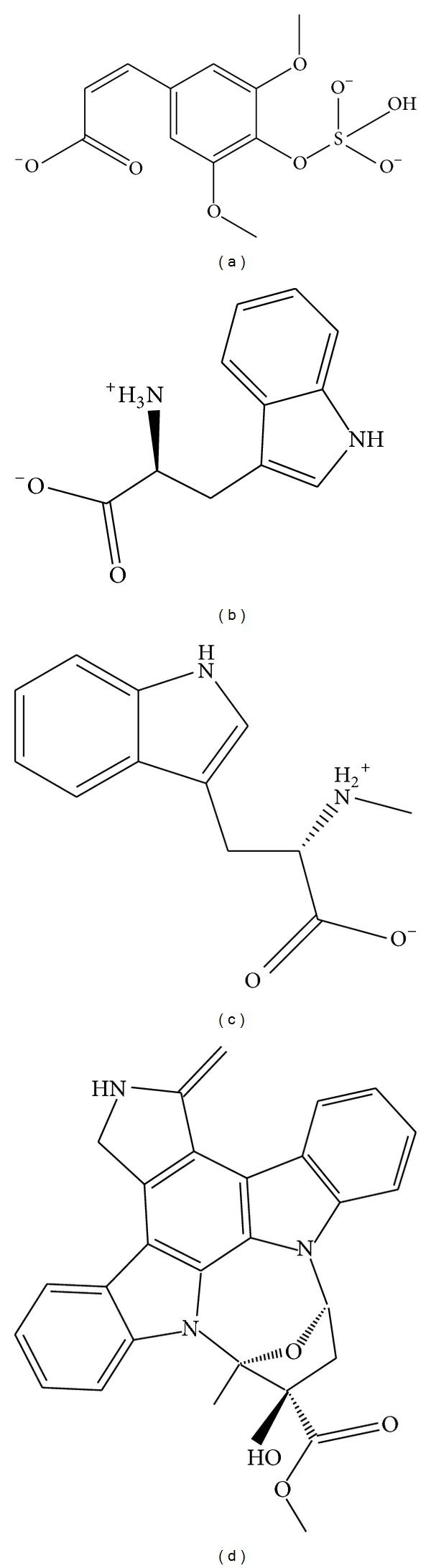
The scaffold of top three TCM compounds and control: (a) sinapic acid-4-O-sulfate (b), kaempferol (c), alpha-terpineol (d), and staurosporine.

**Figure 2:**
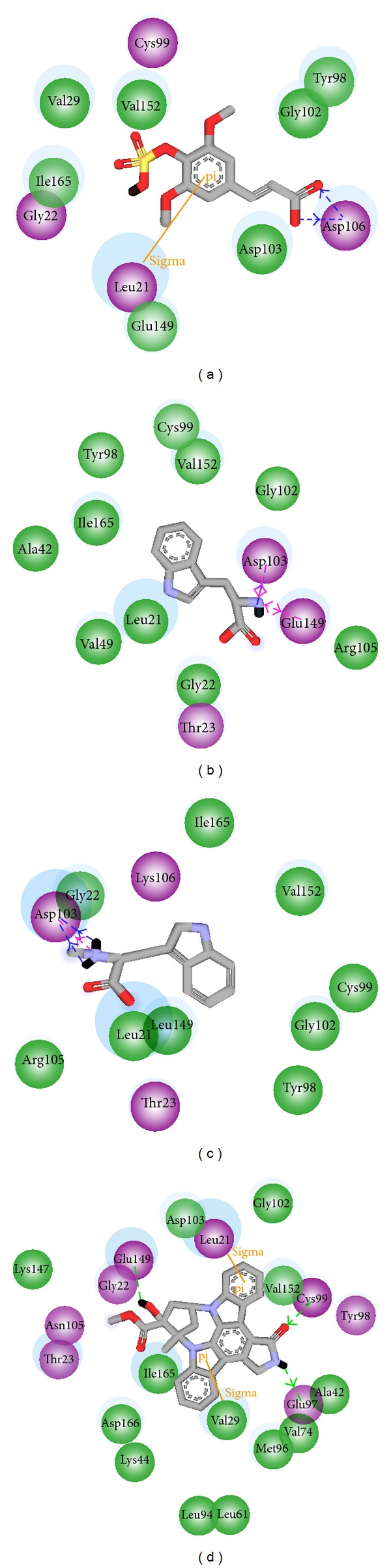
The ligands and poses of docking site for IKK2 and its docking site crystal structure: (a) sinapic acid-4-O-sulfate, (b) kaempferol, (c) alpha-terpineol, and (d) staurosporine.

The candidate compounds and control structure were selected after screening the TCM database (Figure 1). After that, the amino acid neighbors by ligand docking site were displayed in Figure 2. We found Asp103, Leu21, and Cys99 are amino acids that could interact with control and selected compound ligands. We considered these three amino acids as playing a crucial role with regard to IKK2 target function.

**Figure 3:**
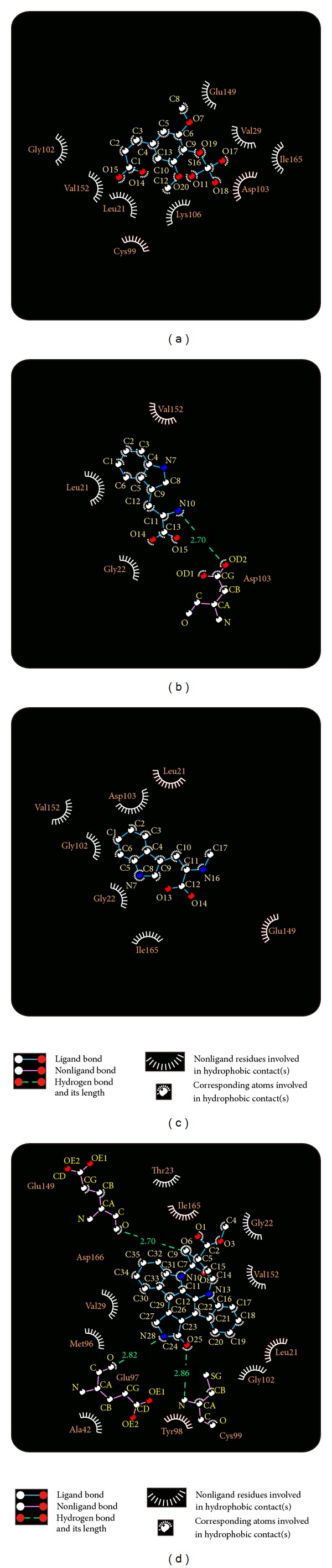
Protein-ligand interactions by Ligplot plus: (a) sinapic acid-4-O-sulfate, (b) kaempferol, (c) alpha-terpineol, and (d) staurosporine. The high frequency hydrophobic interactions, ligands' interactions, were showed by deep red color spots.

The Ligplot plus [66] applied for hydrophobic interaction ligand-compound complex survey (Figure 3). The amino acids Glu149, Val29, Ile165, Val152, Gly102, and Gly22 presented deep red color showing high frequency protein-ligand interactions by hydrogen bond or hydrophobic interactions. These amino acids are very crucial for reference and we selected compounds which were proven by hydrophobic interaction analyses that have an effect on IKK2.

### 3.2. The Results of Disorder Proteins Detection

**Figure 4:**
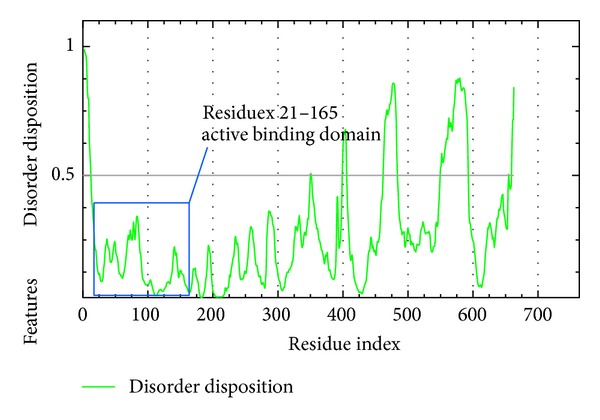
PONDR-FIT prediction of IKK2 active binding domain, value of disorder disposition below 0.5 indicated order residues.

There were important amino acids nearby the docking site for IKK2 docking which include the Asp103, Leu21, Cys99, Glu149, Val29, Ile165, Val152, Gly102, and Gly22. They were considered as active ATP binding sites in IKK2. Staurosporine can inhibit IKK2 by these regions through the binding of targets and as a reference to other compounds. Disorder prediction shows that the binding residues 21 to 165 are all located in ordered region below 0.5 disorder disposition as showed in Figure 4.

### 3.3. Simulation for Molecular Dynamics

**Figure 5:**
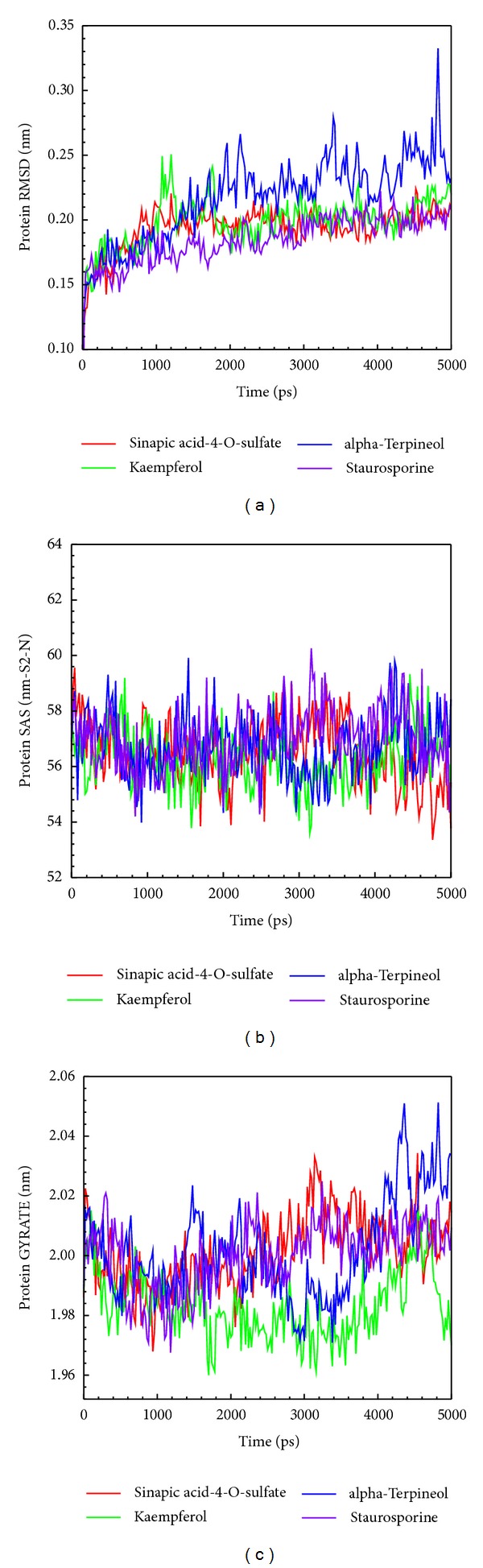
Trajectories of (a) complex RMSD, (b) SASA, and (c) gyrate.

The trajectory of protein-ligand complexes were calculated during MD simulation (Figure 5). All of the three top compounds, sinapic acid-4-O-sulfate kaempferol, and alpha-terpineol, with low variation showed in the complex RMSD for the IKK2 phosphorylation interaction. The stable distance tended to be 0.25 nm of the complex RMSD showing a stable and balanced complex interaction. SASA analysis showing that the solvent area revealed a stable in IKK2 after the three compounds bindings in Figure 5(b). Gyration displayed a stable fluctuation between 1.96 and 2.04 nm during the IKK2 bindings to each of the three compounds during the MD simulation.

**Figure 6:**
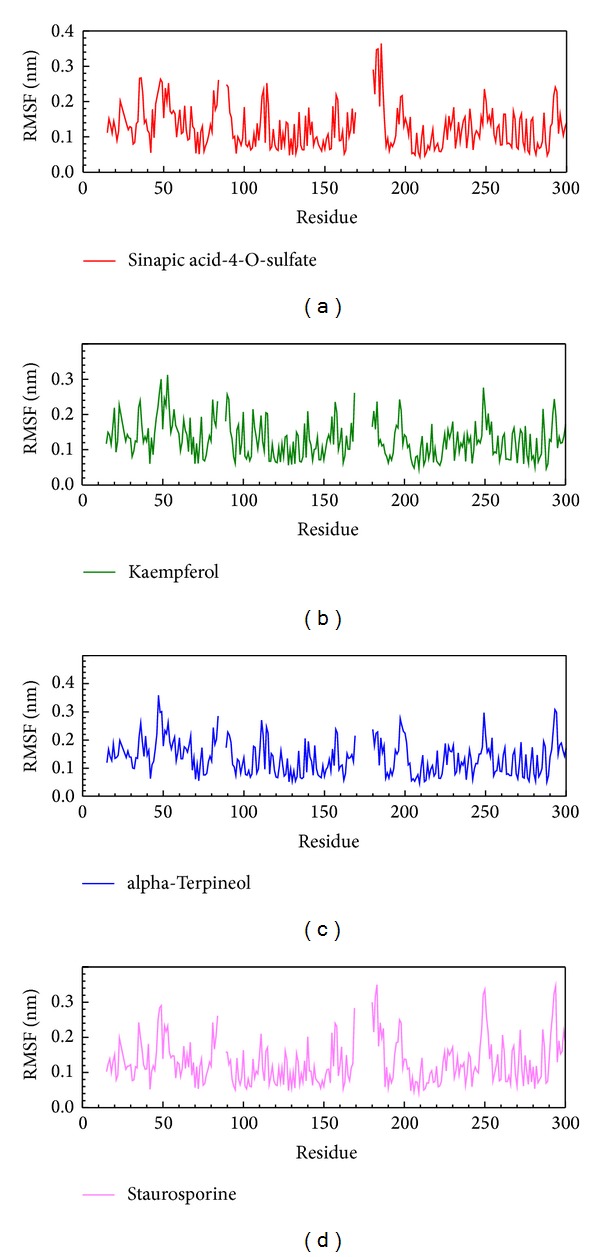
The variation of staurosporine and IKK2 complex in RMSF MD simulation: (a) sinapic acid-4-O-sulfate, (b) kaempferol, (c) alpha-terpineol, and (d) staurosporine.

**Figure 7:**
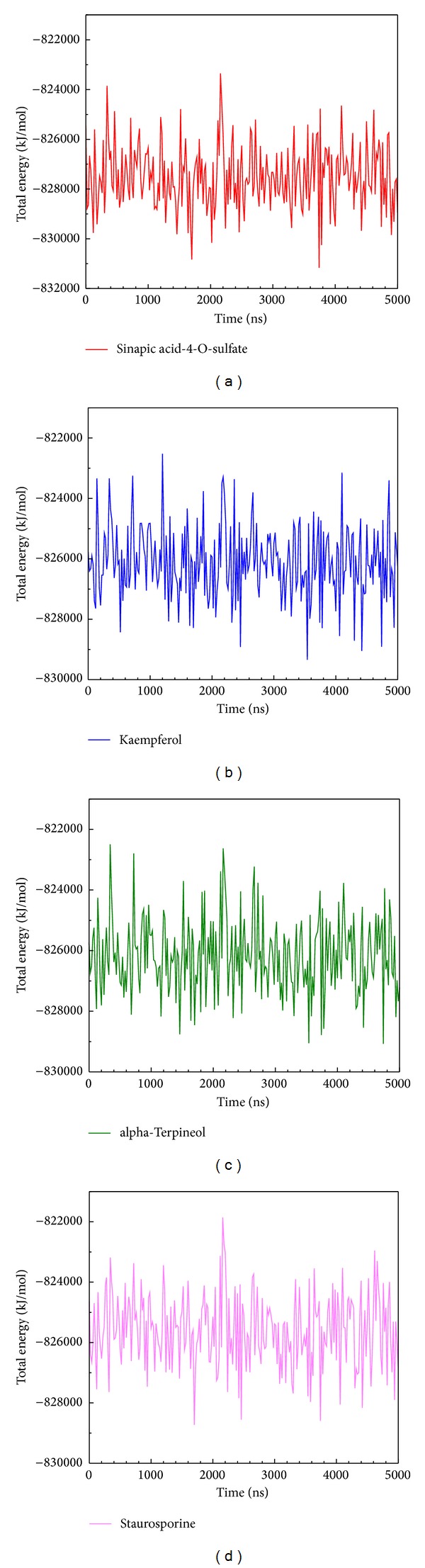
The MD simulation showed the binding energy variation of top three compounds and control to IKK2 complex: (a) sinapic acid-4-O-sulfate, (b) kaempferol, (c) alpha-terpineol, and (d) staurosporine.

In stability analysis of each residue on the binding region over MD simulation, the major binding regions are located on 21 to 149 residues (Figure 6), and we found that the RMSF values are not substantial fluctuations and are similar to each other, exhibiting stable conformation during MD simulation. The trajectories of total energy by MD measurement are presented in Figure 7 which shows that the three top compounds and the staurosporine binding to form IKK2 complex have no significant difference in total energy compared to each other. The ranges of total energy were between −822000 and −820000 kJ/mol and stabilized to −868000 kJ/mol. This presented that the complex is still in a stable still interaction even after a 5000 ps as showed in Figure 7.

**Figure 8:**
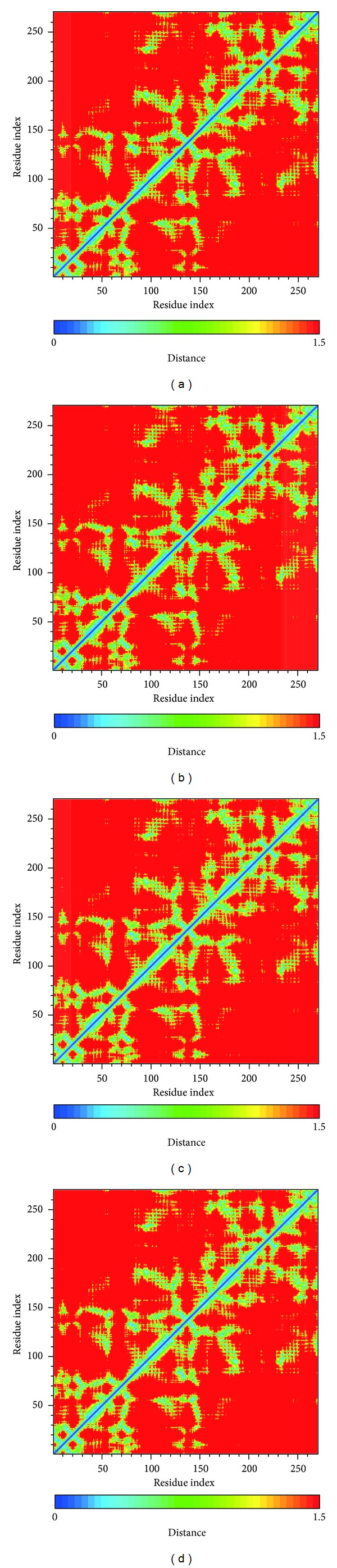
The residue index of bindings distances change: (a) sinapic acid-4-O-sulfate, (b) kaempferol, (c) alpha-terpineol, and (d) staurosporine.

**Figure 9:**
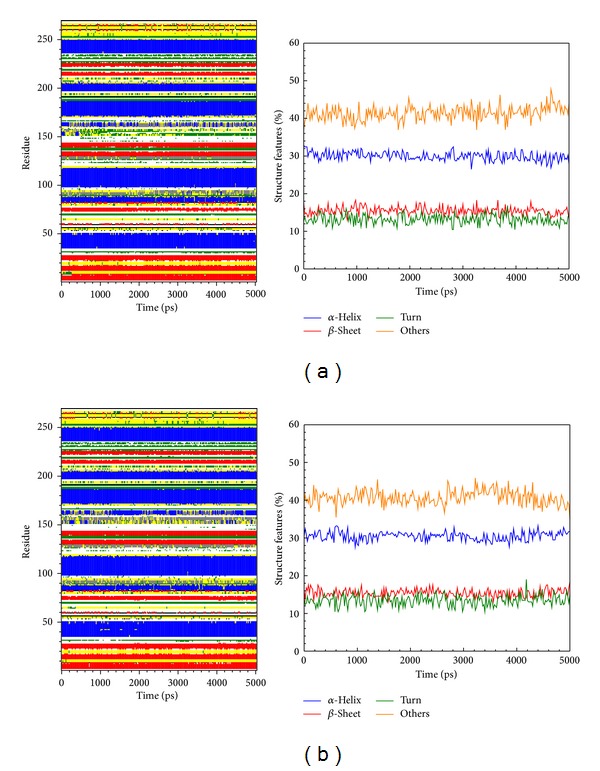
The variation of secondary structural changes during binding process showed by MD simulation of top candidates to IKK2: (a) sinapic acid-4-O-sulfate and (b) kaempferol.

**Figure 10:**
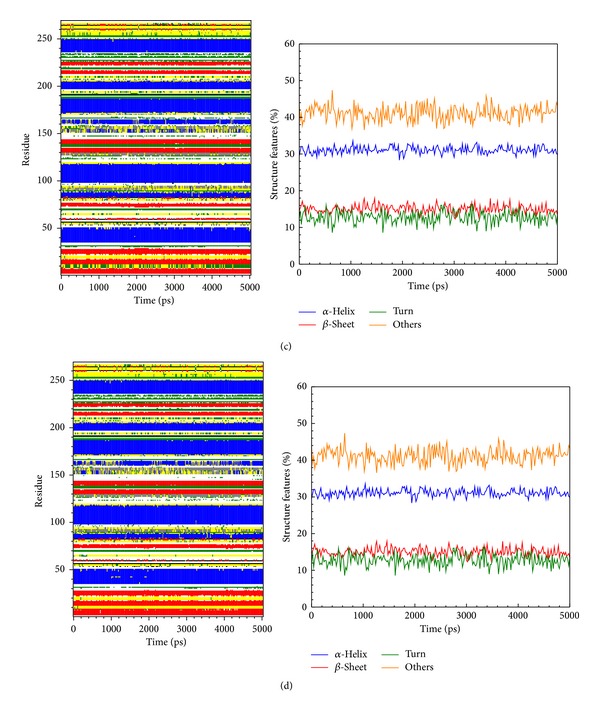
The variation of secondary structural changes during binding process showed by MD simulation of top candidate and control to IKK2: (c) alpha-terpineol and (d) staurosporine.

We calculated distance for pair of each residue during all simulation time; there is no significant difference between all protein-ligand complexes, indicating that complexes remain stable during the simulation time (Figure 8). In DSSP analysis (Figures 9 and 10), the MD simulation showed IKK2 as having three different binding phases. The IKK2 is a transmembrane protein with no change in the structure (helix becoming a loop or others) of the three compounds and staurosporine when binding to IKK2. In Figures 9 and 10, we found these three compounds were all potent candidates for the treatment of COPD patients and all the compounds and control did not have their structure composition broken and had a stable complex interaction.

In addition, the distance of H-bond affecting the occupancy of H-bond was also calculated during MD (Table 2). We found the important amino acids Asp103 and Lys106 having high occupancy H-bond formation between IKK2 phosphorylation sides and sinapic acid-4-O-sulfate and kaempferol. This may explain why these amino acids are important as they act on IKK2 phosphorylation and cause inhibition of NF-KB related inflammation.

**Figure 11:**
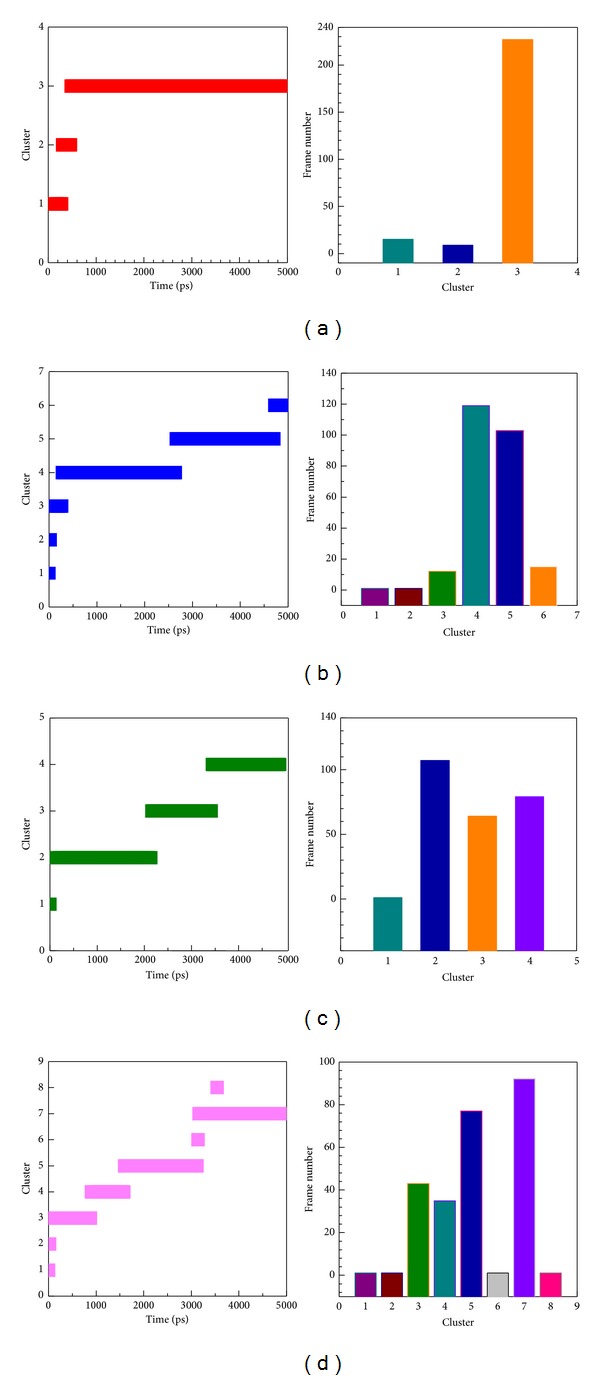
Further clustering analysis of the hit component for further IKK2 binding MD survey.

**Figure 12:**
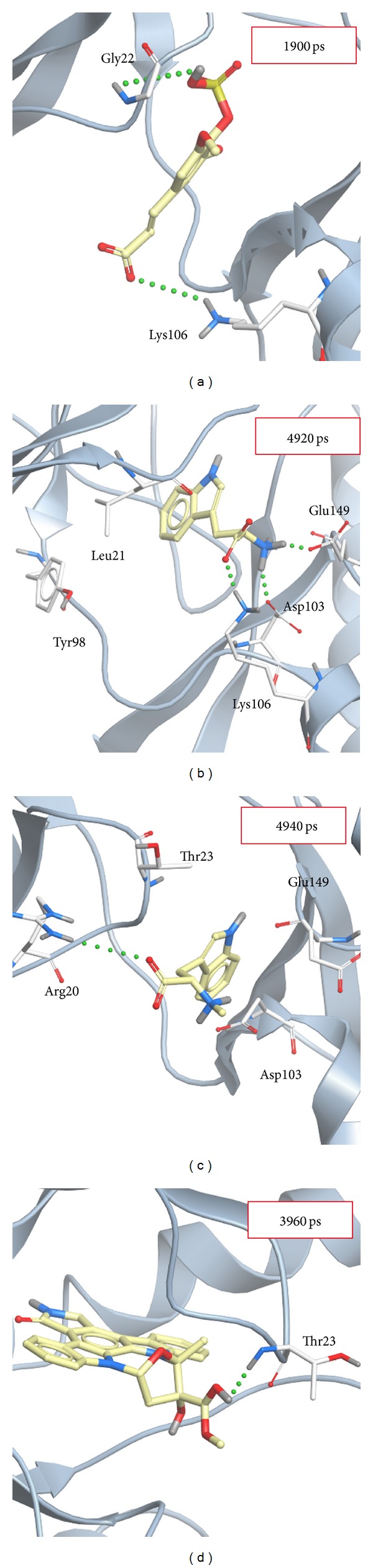
Snapshots of initial conformation and represented structures: (a) sinapic acid-4-O-sulfate, (b) kaempferol, (c) alpha-terpineol, and (d) staurosporine.

After that, we clustered all MD structures using linkage algorithm to identify represented conformation for interaction analysis. All MD frames of four complexes with docked ligand were clustered to different groups (Figure 11), and the middle structures of each group are represented in Table 3. We selected the largest group in latest MD time as represented structure for snapshots study. In Figure 12(a), snapshots of sinapic acid-4-O-sulfate forms H-bond interactions with Gly22 and Lys106.  Figure 12(b) presented kaempferol forms H-bond interaction with Asp 103, Lys106, and Glu149.  Figure 12(c) showed alpha-terpineol forms H-bond interaction with Arg20.  Figure 12(d) revealed that staurosporine generates H-bond interaction with Thr23. Comparing with initial docking pose of sinapic acid-4-O-sulfate revealed H-bond interaction with the same Lys106 showed in Figure 2(a). Therefore, there is the same residue after MD simulation for sinapic acid-4-O-sulfate.

**Figure 13:**
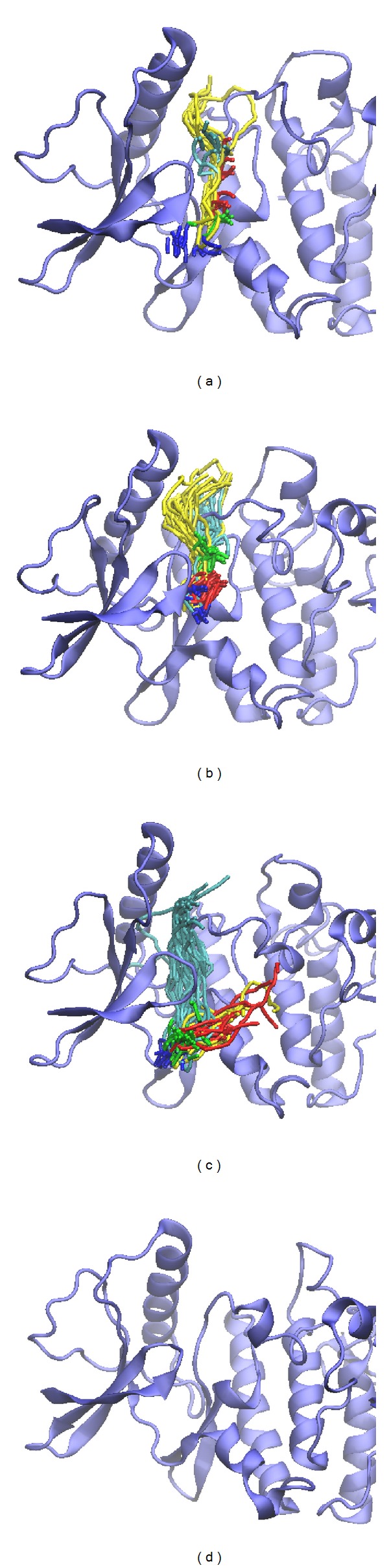
Prediction of ligand channels of the hit component during MD simulation.

For docking pose of kaempferol in Figure 2(b), Glu149 and Asp103 were the initial binding residues. However, only Asp103 keeps the same stable residue in the H-bond interaction after MD simulation. It is worthy to notice that the initial binding poses of alpha-terpineol and staurosporine are different after MD simulation showed in Figures 3(c) and 3(d). The results suggest sinapic acid-4-O-sulfate and kaempferol are more potent compounds candidate for IKK2 interaction. Besides, we further analyzed the ligand pathway during MD simulation. The prediction results were presented in Figure 13; we found that (a) sinapic acid-4-O-sulfate, (b) kaempferol, and (c) alpha-terpineol all have ligand channels. However, there was no ligand path for staurosporine; it is possible that the staurosporine is too compact to generate a ligand channel. We considered that these three compounds could really interact with IKK2 and consequently affect the related function of IKK2 during the binding process.

By TCM targeting IKK2 when drug screening, we found the three compounds from Chinese medicine to treat the COPD and believe that this may help clinicians select potent medicine to prevent patients from having COPD in the future or to assist in the area of disease control for COPD. This identification method can also be useful for many other infectious or inflammatory diseases in terms of selecting the proper drugs for difficult treating diseases.

## 4. Conclusion

Based on the above discussion, we identified the top 3 TCM compounds, sinapic acid-4-O-sulfate, kaempferol, and alpha-terpineol, which can have an effect on IKK2 inhibition and prevent exacerbation and disease progression with regards to COPD. Asp103, Leu21, Cys99, Glu149, Cal29, Val152, Gly22, and Gly102 108 present their crucial effect on IKK2 inhibition through H-bond formation and hydrophobic interaction. The Asp103 and Lys106 are very important residues in IKK2 binding. These top three compounds can bind to the IKK2 ATP binding site and cause IKK2 inhibition by phosphorylation and may be used in future considerations in the development of novel therapies for COPD.

## Figures and Tables

**Table 1:** Scoring functions of top high ranking candidates from docking results.

Name	Dock Score	-PLP1	-PLP2	-PMF
Sinapic acid-4-O-sulfate	189.61	62	60.93	48.9
Kaempferol	174.852	54	47.17	44.31
*α*-Terpineol	157.438	39	33.49	25.96
Geranial	150.458	42	43.71	22.91
3-(2-Carboxyphenyl)-4(3H)-quinazolinone	147.919	54	46.9	33.24
Arctigenin	145.065	71	72.46	85.33
Notoginsenoside G	143.481	54	49.1	38.14
*Staurosporine	**115.865**	**141**	**129.69**	**117.56**

*Control.

**Table 2:** H-bond occupancy among all MD simulations.

Compound name	Atoms of H-bonds	Occupancy	Compound name	Atoms of H-bonds	Occupancy
Sinapic acid-4-O-sulfate	O14:LIG—NZ:LYS106	10.76%	Kaempferol	HZ3:LYS106—O14:LIG	18.33%
	HZ3:LYS106—O15:LIG	42.23%		HZ3:LYS106—O15:LIG	59.76%
	O7:LIG—N:GLY22	0.40%		HN:ASP103—O15:LIG	48.21%
	HN:GLY22—O17:LIG	0.40%		HH:TYR98—O14:LIG	59.36%
	O18:LIG—N:GLY22	5.58%		H27:LIG—O:LEU21	23.51%
	HN:GLY22—O20:LIG	12.35%		H27:LIG—OD1:ASP103	74.90%
	—	—		H27:LIG—OD2:ASP103	47.01%
	—	—		H27:LIG—OE2:GLU149	9.16%
	—	—		H27:LIG—O:GLU149	14.34%

*alpha*-Terpineol	HG1:THR23—O13:LIG	29.48%	Staurosporine	O25:LIG—N:THR23	0.00%
	HG1:THR23—O14:LIG	51.00%		HN:THR23—O1:LIG	59.36%
	HH22:ARG20—O13:LIG	63.35%		O:GLU97—O6:LIG	0.00%
	HH22:ARG20—O14:LIG	54.58%		—	—
	HH12:ARG20—O13:LIG	55.38%		—	—
	HH12:ARG20—O14:LIG	47.01%		—	—
	HE:ARG20—O13:LIG	2.79%		—	—
	HE:ARG20—O14:LIG	4.38%		—	—
	H30:LIG—OD1:ASP103	3.98%		—	—
	H30:LIG—OD2:ASP103	3.19%		—	—
	H22:LIG—OE1:GLU149	15.54%		—	—
	H22:LIG—OE2:GLU149	16.33%		—	—

*LIG: ligand.

**Table 3:** Time of middle frames in each cluster.

Cluster	Time of middle frame (ps)			
	Sinapic acid-4-O-sulfate	Kaempferol	*alpha*-Terpineol	Staurosporine
1	200	0	0	0
2	380	20	780	20
3	1900	140	2840	520
4	—	1320	4940	1280
5	—	3620	—	2460
6	—	4920	—	3140
7	—	—	—	3960
8	—	—	—	3540
